# Role of a corrugated Dion–Jacobson 2D perovskite as an additive in 3D MAPbBr_3_ perovskite-based light emitting diodes†

**DOI:** 10.1039/d2na00942k

**Published:** 2023-03-29

**Authors:** C. T. Prontera, D. Taurino, A. Coriolano, A. Maggiore, M. Pugliese, R. Giannuzzi, F. Mariano, S. Carallo, A. Rizzo, G. Gigli, L. De Marco, V. Maiorano

**Affiliations:** a CNR NANOTEC—Institute of Nanotechnology c/o Campus Ecotekne, University of Salento Via Monteroni 73100 Lecce Italy tania.prontera@nanotec.cnr.it luisa.demarco@nanotec.cnr.it; b Department of Mathematics and Physics “Ennio De Giorgi”, University of Salento 73100 Lecce Italy

## Abstract

Metal halide perovskites represent an intriguing class of materials, and a very promising approach to tune the properties of optoelectronic devices and improve their performance involves the implementation of architectures based on mixed 3D and 2D perovskites. In this work, we investigated the use of a corrugated 2D Dion–Jacobson perovskite as an additive to a classical 3D MAPbBr_3_ perovskite for applications in light-emitting diodes. Taking advantage of the properties of this emerging class of materials, we studied the effect of a 2D 2-(dimethylamino)ethylamine (DMEN)-based perovskite on the morphological, photophysical, and optoelectronic properties of 3D perovskite thin films. We used α-DMEN perovskite both in a mixture with MAPbBr_3_ creating mixed 2D/3D phases and as a passivating thin layer deposited on the top of a 3D perovskite polycrystalline film. We observed a beneficial modulation of the thin film surface, a blue shift in the emission spectrum, and enhanced device performance.

## Abstract 2

A corrugated Dion–Jacobson 2D perovskite has been integrated with a 3D MAPbBr_3_ perovskite by using a blend and bilayer approach for application in light emitting diodes.

## Graphical abstract

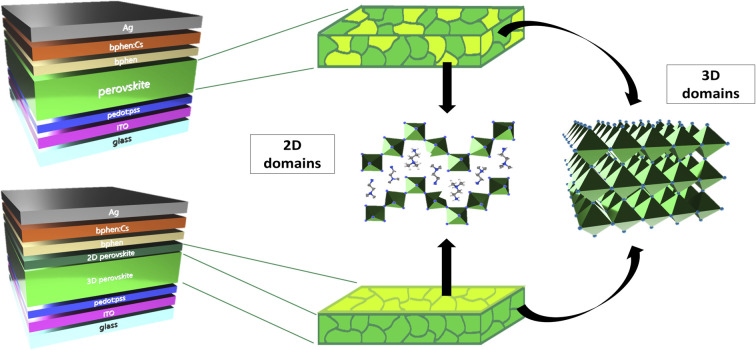

## 1. Introduction

Metal halide perovskites have revolutionized the field of 3rd generation photovoltaics,^1^ providing a new class of promising materials for different optoelectronic devices as well.^2,3^ Their excellent photophysical properties, such as narrow emission with high photoluminescence quantum yields (PLQY), widely tunable light emitting color and high carrier mobility, combined with convenient solution processability, make them attractive in particular for light-emitting diodes (LEDs).^3–9^

Studies on the use of perovskites for optoelectronic applications have been carried out since 1994. However, the first interesting results for the LED application came out only in 2014, demonstrating the possibility of obtaining high-brightness light emitting diodes in the near-infrared, red and green parts of the spectrum by using mixed halide 3D perovskites (MAPbI_3–*x*_Cl_*x*_ (MA = methylammonium)) and solution-based deposition.^10^ These three-dimensional (3D) perovskites have a general formula of AMX_3_ and a cubic unit cell (where A is a monovalent cation that could be organic, such as in the case of methylammonium (MA), and/or inorganic, such as cesium; M is a divalent metallic compound, usually Pb^2+^; and X is a monovalent halide anion such as I^−^, Br^−^, or Cl^−^).^11^ Noteworthily, external quantum efficiencies (EQEs) higher than 20% were successively reached in 3D perovskite based LEDs.^8,12,13^ However, these systems suffer from facile dissociation of excitons due to their long diffusion length^14,15^ and low exciton binding energy^16,17^ that could decrease device performances.^18,19^ It has been demonstrated that a small exciton diffusion length can be achieved by reducing the grain size, thus inducing an improvement of device characteristics.^20^ This effect can be obtained by substituting small cations (MA or cesium) with larger organic cations. These cations act as spacers in the 3D network which, thanks to the formation of such isolated octahedral layer patterns, lead to the formation of quantum well super-lattices.^21,22^ In contrast to the bulky 3D crystalline structures, where the exciton is delocalized, the quantum confinement in 2D perovskites leads to a large binding exciton energy that is favorable for radiative recombination and leads to a high PLQY.

Based on these considerations, researchers moved towards the development of 2D perovskites that have been shown to exhibit higher stability allowing LED performances to be improved.^23^ The larger organic cations used in 2D perovskites, as an alternative to the classic MA and cesium cations, present hydrophobic properties that also allow the isolation of the inorganic octahedron from the ambient water and thus the improvement of their moisture resistance and long-term stability under ambient conditions.^24–26^ 2D perovskites are also interesting thanks to the possibility of widely tuning their optoelectronic properties by using different types of large organic cations.^6,22^ Finally, it is worth noting that the addition of 2D components to 3D perovskites is considered an interesting strategy to passivate defects of 3D perovskite thin films and thus to improve the optoelectronic properties.^27,28^

In addition, recently, a new 2D perovskite class has emerged, in which the monovalent organic ammonium cations are substituted by a single bivalent ammonium cation. This results in the formation of a specific class of 2D perovskites, called the Dion–Jacobson (DJ) phase, with the general formula ((A′)(A)_*n*−1_M_*n*_X_3*n*+1_), where A′ is a bidentate large cation between the perovskite layers, A is a small cation (*e.g.* MA), M is a divalent metal ion, which is commonly a lead or tin cation (Pb^2+^ or Sn^2+^), and X represents a halide anion.^29^ Differently from classic Ruddlesden–Popper (RP) 2D perovskites (where A′ is a monodentate large cation), the divalent spacer cation eliminates the weak van der Waals interactions between alternating inorganic layers, leaving only strong hydrogen bonding interactions.^30,31^ This leads to a more stable framework and a shorter interlayer distance than in their RP phase analogs, reducing the barrier to charge transport in their organic layers.^29^ In addition, the higher rigidity of DJ perovskites, compared with their RP counterparts, allows an intensified octahedral distortion to be obtained.^32^ In some cases, the insertion of sterically bulky cations can induce a further distortion of the materials' lattice and variations in their optical and electrical properties.^33^ In particular, some cations can induce the formation of a (110)-oriented corrugated 2D perovskite, which is different from the classic flat perovskite sheets of the (100)-oriented lattice. The distorted nature of these corrugated perovskites generates a broadband photoluminescence, probably related to self-trapped exciton states. As this broad emission covers the entire visible spectrum (white emission), the corrugated perovskites have also been widely studied for lighting applications.^6,33,34^

In this work, we exploited the interesting properties of a corrugated Dion–Jacobson 2D perovskite, namely α-(DMEN)PbBr_4_ (DMEN: 2-(dimethylamino)ethylamine),^33^ by properly combining it with a 3D MAPbBr_3_ perovskite. Mixed architectures based on 3D and 2D perovskites have sometimes been used as a tool to improve the optoelectronic properties of LEDs, particularly those based on RP perovskites.^13,35,36^ In contrast, the application of DJ perovskites in this field is less common,^37–39^ and in particular, to the best of our knowledge, the effect of a corrugated one on the morphological, photophysical and optoelectronic properties of perovskite thin films and on LED performances has never been studied.

We integrated α-(DMEN)PbBr_4_ by using two different approaches: (i) the 2D precursors were mixed with the 3D precursors in different ratios; (ii) a thin layer of 2D perovskite was spin coated on top of the 3D polycrystalline film. We then observed that it is possible to advantageously modulate the light-emitting properties of the perovskite active layer and induce passivation of the defects in the polycrystalline film, resulting in improved LED performance.

## 2. Experimental part

### 2.1 Materials

Lead(ii) bromide (PbBr_2_) and hydrobromic acid (HBr) were purchased from Alfa Aesar. Dimethyl sulfoxide (DMSO), *N*,*N-*dimethylformamide (DMF), tetrahydrofuran (THF), ethanol (EtOH), 2-(dimethylamino)ethylamine (DMEN), diethyl ether (DE), toluene, bathophenanthroline (BPhen) and Ag where purchased from Sigma Aldrich. Methylammonium bromide (MABr) was purchased from LUMTEC. Clevios AI4083 was purchased from HeraeusCleviosGmbH, and was used as HIL. Cesium was supplied by Saes Getters. All chemicals were used as received without any further purification. ITO (Indium Tin Oxide) covered glass substrates were supplied by VisionTek Systems Ltd and used as an anode layer.

#### Synthesis of α-(DMEN) salt

3.4 ml of 47% wt HBr was added to 5 ml of EtOH by heating under stirring at 80 °C. Then 2.2 ml of DMEN (20 mmol) was added dropwise to the previous mixture. The solution was kept stirring at 80 °C for 4 hours. Then the resulting yellow precipitate was washed with diethyl ether. The procedure was repeated several times until a white powder was obtained, which was finally dried in a vacuum for 12 h.

### 2.2 Perovskite thin films preparation

The pure 3D thin film was prepared starting from a 0.5 M or 0.25 M PbBr_2_ and MABr (molar ratio 1 : 1.4) blend dissolved in DMF and DMSO in a 4 : 1 volume ratio.

Blend samples were prepared from solutions of PbBr_2_, MABr and α-(DMEN) salt dissolved in DMF and DMSO (4 : 1) according to the molar ratios.

Perovskite thin films were obtained by spin coating the precursor solutions in a nitrogen filled glovebox, by using a two-step deposition process (500 rpm for 5 s and 2000 rpm for 30 seconds). Toluene was dropped on the film 10 seconds from the end of the spin coating process. The samples were then annealed at 110 °C for 10 minutes. Thin films of about 30–40 nm were obtained with the 0.25 M solutions, while thicknesses of about 50–60 nm were obtained with the 0.5 M solutions.

For the optical and morphological characterization, films obtained starting from 0.5 M solutions were employed, while both thin film thicknesses were tested as emitting layers of LED architectures.

The 3D/2D bilayers were obtained starting from a standard 3D thin film. A 2D precursor solution in THF (0.01 M) was spin coated in a N_2_-filled glovebox on top of the 3D thin film by using a 2 step deposition process (500 rpm for 5 s and 2000 rpm for 30 seconds) and annealed at 60 °C for 5 minutes (no dripping was performed).

### 2.3 Perovskite thin films characterization

The absorbance spectra of the perovskite thin films were measured using a PerkinElmer UV/Vis/NIR spectrometer (Lambda 1050).

Uncorrected emission spectra were obtained with an Edinburgh FLS980 spectrometer equipped with a Peltier-cooled Hamamatsu R928 photomultiplier tube (185–850 nm). Corrected spectra were obtained *via* a calibration curve supplied with the instrument.

Emission lifetimes in the ps to μs range were determined with the single photon counting technique by means of the same Edinburgh FLS980 spectrometer using a laser diode as the excitation source (1 MHz, *λ*_exc_ = 407 nm) and a Peltier-cooled Hamamatsu R928 photomultiplier tube as the detector. Analysis of the luminescence decay profiles *vs.* time was accomplished with the DAS6 decay analysis software provided by the manufacturer.

For all solid films, photoluminescence quantum yield (PLQY, *Φ*_PL_) was calculated using the corrected emission spectra obtained from an apparatus consisting of a barium sulphate coated integrating sphere (4 or 6 inches) and a 450 W Xe lamp (*λ*_exc_ = tunable by a monochromator supplied with the instrument) as light sources, and an R928 photomultiplier tube as a signal detector, following the procedure described by De Mello *et al.*^40^ Experimental uncertainties are estimated to be ±8% for lifetime determinations, ±20% for emission quantum yields, and ±2 nm and ±5 nm for absorption and emission peaks, respectively.

The morphology of the thin films was evaluated *via* scanning electron microscopy (Zeiss FE-SEM Merlin).

### 2.4 Perovskite LEDs fabrication and characterization

The LED architecture is as follows: glass/ITO/PEDOT:PSS (30 nm)/perovskite (30–40 nm or 50–60 nm)/BPhen (10 nm)/BPhen:Cs (40 nm)/Ag (100 nm).

The hole transport layer and the emitting layer were spin coated (PEDOT:PSS in air and emitting layer in a glovebox with nitrogen) and the hole blocking layer, electron transport layer and anode were deposited *via* thermal evaporation in a Kurt J. Lesker multiple high-vacuum chamber system.

The optoelectronic characteristics of the OLED devices were measured in a glovebox using an Optronics OL770 spectrometer coupled with an OL610 telescope unit with an optical fiber for the luminance measurements. The whole system was calibrated by the National Institute of Standards and Technology (NIST) using a standard lamp, and was directly connected by an RS232 cable to a Keithley 2420 current–voltage sourcemeter.

The operational stability of the devices was measured in a N_2_-filled glovebox. The luminance value at 7 V was monitored with an interval time of 10 s using the above-mentioned instrumentation.

## 3. Results and discussion

With the aim of investigating the effect of a corrugated DJ 2D perovskite precursor as an additive in MAPbBr_3_, four different blends were prepared with an increasing amount of α-DMEN salt at the expense of MABr, which for simplicity will be indicated as N12, N7, N5 and N3 as shown in Table 1.

**Table 1:** Perovskite precursor solutions composition^a^

Sample	m.r. of PbBr_2_	m.r. of α-(DMEN) salt	m.r. of MABr	Total m.r.
3D	1	0	1.4	1 : 0 : 1.4
N12	1	0.1	1.3	1 : 0.1 : 1.3
N7	1	0.2	1.2	1 : 0.2 : 1.2
N5	1	0.3	1.1	1 : 0.3 : 1.1
N3	1	0.4	1	1 : 0.4 : 1

a m.r. = molar ratio.

**Fig. 1:**
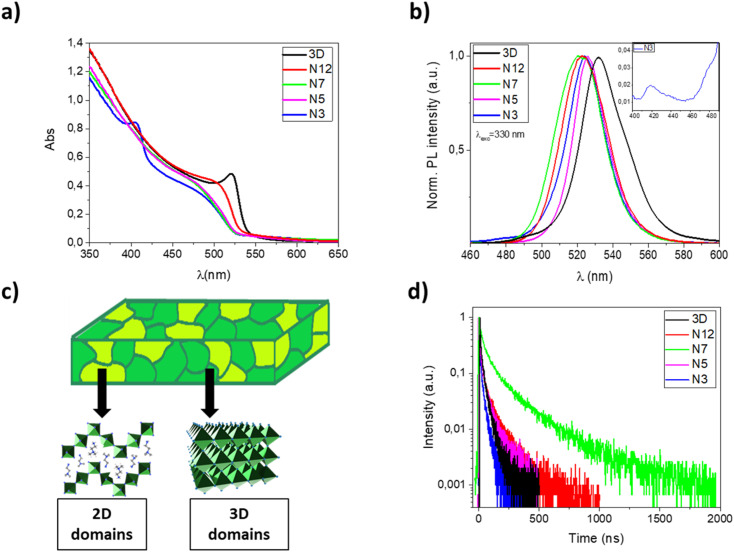
(a) UV-vis absorption spectra of 3D, N12, N7, N5 and N3 thin films on glass substrates; (b) PL spectra of 3D, N12, N7, N5 and N3 thin films on glass substrates; (c) schematic of the 2D–3D perovskite thin films with domains at different dimensionality; (d) PL decay of 3D (*λ*_em_ 532 nm), N12 (*λ*_em_ 523 nm), N7 (*λ*_em_ 520 nm), N5 (*λ*_em_ 526 nm) and N3 (*λ*_em_ 524 nm) thin films on glass substrates.

In Fig. 1a and b, UV-Vis absorption and photoluminescence spectra of 3D, N12, N7, N5 and N3 thin films are reported. The pristine MAPbBr_3_ film shows an absorption onset around 540 nm and an emission peak at 531 nm with a full width at half maximum (FWHM) of about 30 nm, which is consistent with literature values.^41^

With the incorporation of α-(DMEN)Br salt, it is possible to observe a progressive blue shift of the absorption onset and a reduction of the absorption intensity with respect to the pure 3D thin film. Indeed, the insertion of α-(DMEN)Br salt induces the formation of low dimensional phases which exhibit blue-shifted absorption in the electromagnetic spectrum with respect to the corresponding 3D phase. In particular, the N3 thin film shows an absorption peak at about 400 nm which is very similar to that reported for the pure 2D thin film,^33^ indicating the presence of low-dimensional phases. In contrast, the absorption spectra of N12, N7 and N5 demonstrate that their dimensionality is only slightly lower than that of the pure 3D phase, while in the case of the N3 sample there is the coexistence of high-dimensional phases with low-dimensional phases, as sketched in Fig. 1c.

Photoluminescence (PL) spectra show a narrow FWHM and small Stokes shift for all samples, as previously observed in the case of perovskites. Interestingly, the emission of thin films obtained from the blends is blue shifted compared to that of the pure 3D perovskite. This is due to the presence of mixed 2D/3D domains. In particular, sample N3 shows emission peaks/shoulders of very low intensity at about 420 and 480 nm (see Fig. 1b, inset) ascribable to low dimensional phases.

The low intensity of these peaks, compared to the intense absorption peak of the N3 sample at 400 nm, is attributable to an efficient energy funneling from low dimensional phases to 3D and mixed 2D/3D phases resulting in light emission typical of the 3D and blended structures.^13,42^

In order to evaluate the effect of the α-DMEN component as an additive in MAPbBr_3_, we have also performed time-correlated single-photon counting (TCSPC) experiments and calculated the photoluminescence quantum yield (*Φ*_PL_) of the thin films (Table 2). The time-resolved photoluminescence decay traces are reported in Fig. 1d while the lifetimes and *Φ*_PL_ values are reported in Table 2.

**Table 2:** PL decay times and PLQY of the perovskite blend samples

	*λ*_em_ a/nm	*Φ*_PL_ b	*τ*_1_ c (ns)	*τ*_2_ c (ns)	*τ*_av_ d (ns)
3D	532	0.023	16.0 (38.7%)	84.7 (61.3%)	77.4
N12	523	0.018	18.3 (38.4%)	97.5 (61.6%)	89.2
N7	520	0.024	65.1 (38.0%)	275.4 (62.0%)	248.8
N5	526	0.012	20.0 (48.5%)	81.5 (51.5%)	70.0
N3	524	0.009	4.8 (45.3%)	26.3 (54.7%)	23.5

a Emission maxima.

b Absolute photoluminescence quantum yield recorded using the integrating sphere.

c Photoluminescence lifetime.

d Photoluminescence average lifetime.

The emission decay curves (Fig. 1d) follow a biexponential kinetics (Table 2) in each case; this relationship suggests that the PL decay of perovskite films occurs through two pathways. The fast decay is assigned to non-radiative recombination processes that can be due to the presence of defects or energy transfer processes such as trap-assisted recombination; the slow component corresponds to radiative recombination.^43,44^ As the proportion of the α-DMEN component in the 3D precursor is increased up to 0.2 (N12 and N7), the PL lifetimes *τ*_1_ and *τ*_2_ also increase, so the average PL lifetime increases. However, when a large quantity of α-(DMEN)Br is added to the precursor solution (N3 and N5), the PL lifetimes *τ*_1_ and *τ*_2_ decrease becoming even shorter than for the pure 3D sample.

Also, we performed PLQY measurements (Table 2) to compare *Φ*_PL_ of the quasi-2D and 3D perovskite films and we observed a similar trend for the PL lifetime. We can see that if a small amount of the 2D precursor is added (N12 and N7) the *Φ*_PL_ remains almost the same; however, when a larger quantity of 2D perovskite is added to the blend (N3 and N5) the *Φ*_PL_ is reduced by up to more than half with respect to the pure 3D thin film.

The observed trend could be explained as a result of different phenomena, such as: quantum confinement effect related to the formation of quantum well structures, a different distribution of defects, the passivation of defects in the 3D films by the large cations.^27,35,45^ Indeed, on one hand, the defect passivation associated with the presence of mixed 2D/3D phases in thin films makes it possible to reduce the non-radiative recombination and increase the emission lifetime.^46^ On the other hand, a large amount of the α-DMEN component introduces trap states which are considered efficient emission quenchers. Both PLQY values and decay time demonstrate that among our samples, the N7 sample represents the best compromise and that there is a trade-off in terms of passivation of 3D defects and the best amount of corrugated 2D perovskite in the thin film, which if further increased introduces trap states that quench the emission. These results, especially the increase in the PL decay up to hundreds of nanoseconds, demonstrate that in these kinds of systems can be potentially found an optimal quantity of 2D perovskites that enhance the optical properties in the mixed sample.

**Fig. 2:**
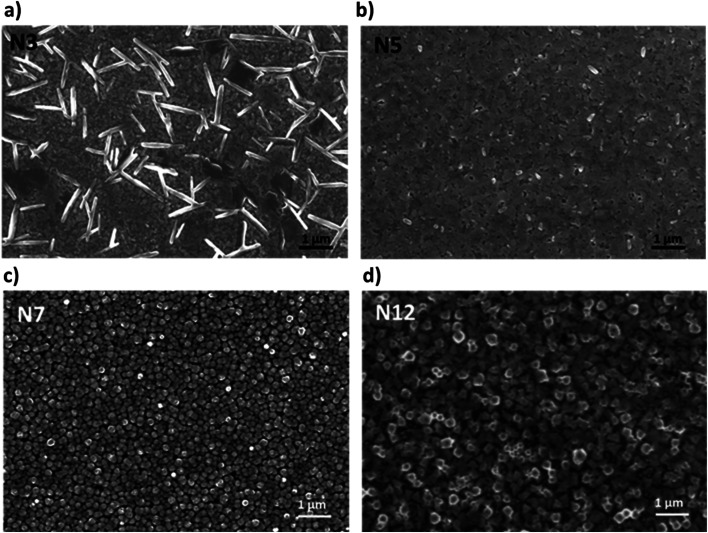
SEM pictures of (a) the N3 sample; (b) N5 sample; (c) N7 sample; and (d) N12 sample.

Film morphology can also be strongly affected by the presence of α-DMEN in the thin films and for this reason it was evaluated through SEM analysis (Fig. 2). The pure 3D film presents the classical 3D perovskite grain structure with dimensions in the order of hundreds of nanometers (Fig. S1†). With the addition of the 2D precursor it is possible to observe a drastic modification of the morphology. N3 shows the presence of lamellar structures embedded in the polycrystalline 3D perovskite film. They are probably related to the higher content of 2D perovskite domains in the thin N3 film (Fig. 2a) and are present in reduced size and number in the N5 sample (Fig. 2b). In contrast, the N7 and N12 samples, in which the 2D content is lower, show a grain structure whose dimensions increase with the reduction of the α-DMEN component (Fig. 2c and d). In particular, the grain size of N7 and N12 is larger than that observed in the pure 3D thin film (Fig. S1†). This effect may be due to an intercalation phenomenon of α-DMEN which can increase the grain dimensions.^47^

**Fig. 3:**
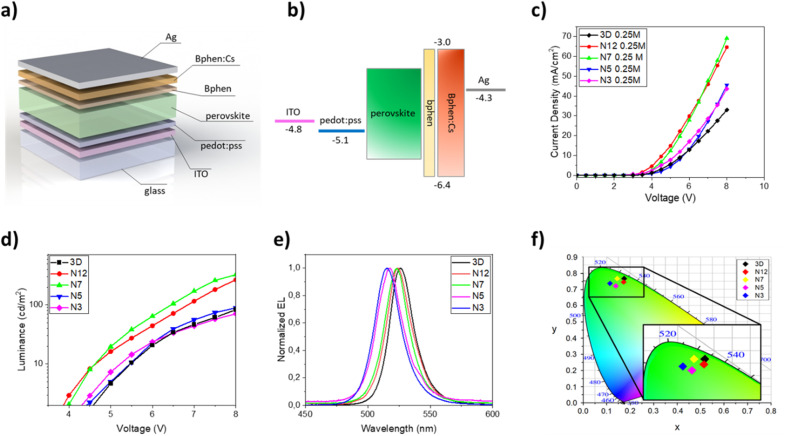
(a) Multilayer architecture of LED devices with the 2D–3D perovskite; (b) band diagram of LED devices with the 2D–3D perovskite; (c) current density *vs.* voltage curves for LED devices obtained with 3D, N12, N7, N5 and N3 thin films (0.25 M); (d) luminance *vs.* voltage curves for LED devices obtained with 3D, N12, N7, N5 and N3 thin films (0.25 M); (e) electroluminescence spectra of LED devices obtained with 3D, N12, N7, N5 and N3 thin films (0.25 M); (f) CIE coordinates plot for LED devices obtained with 3D, N12, N7, N5 and N3 thin films (0.25 M).

Once the optical and morphological characteristics were measured, the mixed 2D/3D thin films were also tested as emitting material for LED architectures. The devices have the following structure: glass/ITO/PEDOT:PSS/perovskite/BPhen/BPhen:Cs/Ag. PEDOT:PSS (30 nm) is used as the hole transport layer (HTL), BPhen (10 nm) is used as the hole blocking layer (HBL), BPhen doped with cesium (40 nm) is used as the electron transport layer (ETL), Ag (100 nm) is used as the anode and the 3D and the mixed 2D/3D perovskites are used as the emitting layer (EL) (Fig. 3a). The band diagram of the device is shown in Fig. 3b. Electro-optical performances of pure 3D and mixed 2D/3D devices prepared by using a 0.25 M precursor (30–40 nm) solution are reported in Fig. 3c–f, while all the data for 0.5 M devices (50–60 nm) are reported in the ESI (Fig. S2 and Table S1†).

The electroluminescence peak is located close to the wavelength of the PL peak (5–10 nm shift) and is very narrow with a full width at half maximum (FWHM) of about 20 nm, as expected for perovskite LEDs (Fig. 3e).^3^ In particular, we can see that there is a blue shift of the EL peak with the increase of the quantity of the 2D precursor in the solution, indicating a variation from pristine 3D thin film towards a mixed 2D/3D thin film. In the mixed samples no additional emission peaks are observed at higher energy which is attributed to low dimensional phases, indicating the good energy transfer from 2D to 3D phases.^36,39,48^ The CIE coordinate graph confirms the blue shift observed in the electroluminescence spectra (Fig. 3f). The current density–voltage–luminance curves (*J*–*V*–EL) (Fig. 3c and d) and a summary table are also reported (Table 3). As is shown in Fig. 3c, it is possible to observe a shift of the *J–V* curves toward lower voltage values for samples N12 and N7 with respect to 3D. This effect can be attributed to defect passivation which improves the charge injection and transport into the thin films with a consequently more efficient radiative recombination.^49^ The higher luminance values and the lower optical switch-on voltages observed for samples N12 and N7 support this hypothesis (Fig. 3d). With a further increase of the 2D component inside the emissive layer there is a shift of the *J*–*V* curve toward higher voltage and a reduction of the luminance values, thus confirming the formation of trap states in the thin film as already observed in the optical study. Moreover, a worsening of the device characteristics can also occur due to the electrically insulating character of the 2D perovskite which becomes relevant beyond a threshold of the 2D component in the thin film.

**Table 3:** Luminance, current efficiency and EQE of LED devices obtained with 3D, N12, N7, N5 and N3 thin films (0.25 M)

	Lum. (cd A^−1^)	CE (cd A^−1^)	EQE (%)
3D	83 @ 8 V	0.25 @ 8 V	0.07 @ 8 V
N12	267 @ 8 V	0.41 @ 8 V	0.12 @ 8 V
N7	325 @ 8 V	0.47 @ 8 V	0.15 @ 8 V
N5	89 @ 8 V	0.20 @ 8 V	0.07 @ 8 V
N3	71 @ 8 V	0.16 @ 8 V	0.05 @ 8 V

The best electroluminescence performances are observed for the N7 based device, as expected by optical and morphological characteristics, with luminance values that reach almost 350 cd m^−2^ at 8 V, and are four times higher than that observed for the 3D device at the same voltage. We also observe an efficiency value that is double that of the device with only 3D at the same voltage value.

The optical, morphological and electro-optical properties observed in the blend samples can be attributed to the intrinsic nature of 2D perovskites. Indeed, the high exciton binding energy and the typical charge confinement effect induce a weakening of free charge capture leading to increased radiative recombination.^50^ Moreover, an excess of large organic molecules can further passivate the surface defects and fill the gaps between perovskite crystals with an improvement of the grain dimensions^50^ (as confirmed by SEM characterization). On the other hand, low-dimensional perovskites could reduce the electroluminescence efficiency due to an inefficient energy transfer.^51^ Therefore, we prepared different samples to understand how the addition of low-dimensional phases into the 3D film can affect their properties and we found the trade-off between the above-mentioned effects. In particular, we conducted this study by using a peculiar bidentate organic molecule which is able to generate a corrugated DJ 2D perovskite. In this regard, quasi 2D structures based on DJ perovskites show a further advantage due to the bidentate nature of the bulky cation that is able to stabilize the crystal structure through the formation of strong bonds, resulting in a tighter connection between the inorganic layers.^38^ At the same time, the distorted nature of the corrugated perovskites could cause the formation of trap states that justify the limited improvement observed in the study.^33^

To further elucidate the effect of the selected 2D perovskite on the device performances, we used this material as a buffer layer on top of the standard 3D perovskite. Indeed, the use of 2D perovskites as a passivating/buffering layer on top of 3D perovskites to improve the performances of both solar cells and LED devices is widely reported.^27,52–54^

**Fig. 4:**
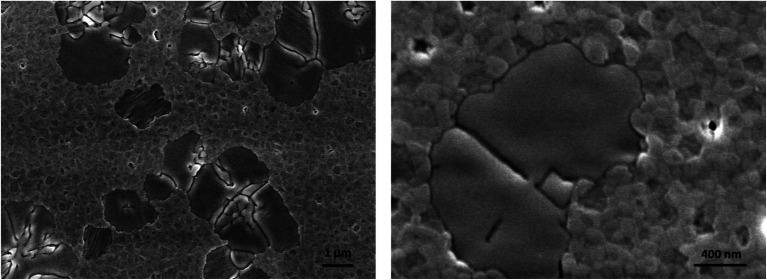
SEM pictures of the 3D/2D bilayer.

Starting from these considerations, we prepared a 3D/2D bilayer by spin coating a dilute solution of the 2D precursors in THF on top of the 3D thin film and it was tested in a LED architecture. THF was chosen to avoid re-dissolution of the underlying perovskite thin film. SEM pictures show that the granular morphology structure of the 3D perovskite is visible together with some flat and smooth islands which can be attributed to the 2D perovskite (Fig. 4). We deliberately chose to use dilute solutions of α-DMEN precursor to avoid the formation of a continuous film of 2D perovskite that would be electrically insulating.

**Fig. 5:**
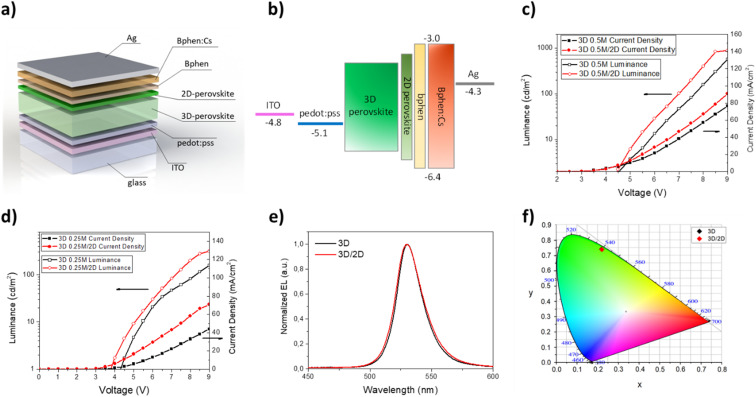
(a) Multilayer architecture of LED devices with the 3D–2D perovskite bilayer; (b) band diagram of LED devices with the 3D–2D perovskite bilayer; (c) current density *vs.* voltage curves for LED devices obtained with the 3D–2D perovskite bilayer at different precursor concentrations (0.25 M and 0.5 M); (d) luminance *vs.* voltage curves for the LED devices obtained with the 3D–2D perovskite bilayer at different precursor concentrations (0.25 M and 0.5 M); (e) electroluminescence spectra of LED devices obtained with the 3D–2D perovskite bilayer; (f) CIE coordinates plot for LED devices obtained with the 3D–2D perovskite bilayer.

The device performances obtained with the 3D/2D bilayer as emitting material are reported in Fig. 5 and they were compared with those observed for the devices with pure 3D. Two different 3D thicknesses were tested by keeping the concentration of the 2D precursor solution constant and the best results were obtained with films obtained starting from a 0.5 M solution (Table 4).

**Table 4:** Luminance, current efficiency and EQE of LED devices obtained with the 3D–2D perovskite bilayer at different precursor concentrations (0.25 M and 0.5 M)

	Lum. (cd A^−1^)	CE (cd A^−1^)	EQE (%)
3D (0.5 M)	573 @ 9 V	0.75 @ 9 V	0.21 @ 9 V
3D (0.5 M)/2D	885 @ 9 V	1.00 @ 9 V	0.27 @ 9 V
3D (0.25 M)	157 @ 9 V	0.36 @ 9 V	0.10 @ 9 V
3D (0.25 M)/2D	317 @ 9 V	0.45 @ 9 V	0.12 @ 9 V

The electroluminescence spectra and the CIE diagram are reported in Fig. 5e and f and no significant differences are observed between the two kinds of devices, demonstrating that the small quantity of the 2D perovskite does not contribute to modifying the emission properties of the 3D thin film.

Regarding the *J*–*V* curves (Fig. 5c), a shift towards lower voltage values is visible for the bilayer sample and it is attributable to a defect passivation effect, as observed for the N12 and N7 samples. Furthermore, the presence of the thin layer of 2D perovskite induces an enhancement of the luminance from about 600 cd m^−2^ (3D) to almost 900 cd m^−2^ at 9 V (0.5 M samples) (Fig. 5d). An enhancement of the current efficiency is also visible from 0.75 cd A^−1^ at 9 V (3D) to 1 cd A^−1^ at 9 V (3D/2D).

**Fig. 6:**
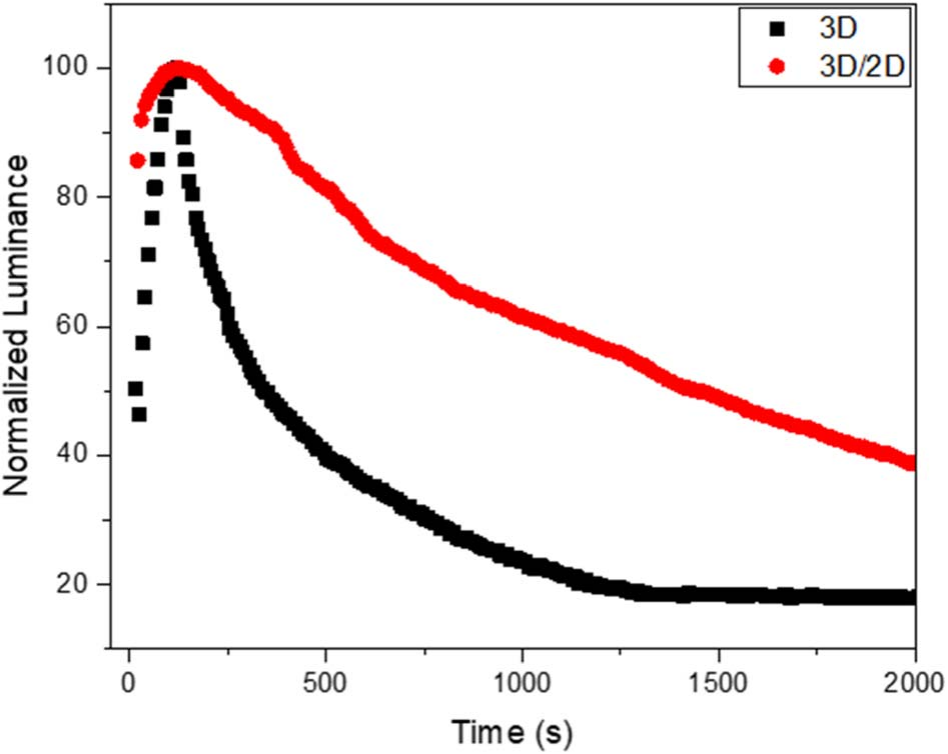
Operational stability of the 3D/2D perovskite-based device and related reference device (3D).

Considering these interesting results, we evaluated the operational stability of the device based on the 3D/2D sample and we compared it with the reference device based on 3D (Fig. 6).

An improvement in the operational stability of the 3D/2D bilayer device compared to the 3D perovskite based device can be readily observed by evaluating the time that the device takes for the luminance to drop to half its initial value during constant applied voltage (T50). Indeed, the bilayer shows a T50 of over 1400 s compared to 340 s for the 3D device.

This behavior could be ascribed to the beneficial presence of the 2D top layer that could suppress interlayer ion migration, mitigating one of the factors of instability of perovskites, namely their intrinsic electrochemical instability, related to ion migration phenomena.^55^ This passivation effect also justifies the higher luminance and efficiency of the bilayer-based device.

The reported results considering the blend approach and the surface approach confirm that the investigated corrugated 2D perovskite can be used to modify the thin film properties of a standard MAPbBr_3_ 3D perovskite thus improving the performances in a LED architecture.

## 4. Conclusion

In this work the passivation effect of a DJ α-DMEN-based 2D perovskite on the optical, morphological, and electro-optical characteristics of a classic MaPbBr_3_ 3D perovskite was studied. Two different approaches were adopted: (i) the 2D precursor was mixed with the 3D precursors in different molar ratios in order to obtain mixed 2D/3D structures; (ii) a thin layer of 2D perovskite was deposited on top of the 3D thin film as a buffer layer.

It was observed that the 2D additive influences the thin film characteristics in different ways. When a low concentration of the 2D component is introduced, a smoother surface, a slight blue shift of the emission spectrum and an increase of radiative decay time with respect to the pure 3D thin film are visible. These changes demonstrate the actual occurrence of defect passivation, which is also confirmed by the electro-optical performance improvement of the corresponding LED devices. With a higher 2D content, a higher trap density is deductible by optical characteristics and a worsening of the electro-optical performance is visible.

In order to determine whether the device performances can be further improved, we tested the possibility of passivating the surface defects of the 3D thin film by spin coating a thin layer of the 2D perovskite on top of it. By using this approach, we observed an increase of the maximum luminance and of the maximum efficiency as well as better performances compared to those obtained with the blend samples.

The results reported in this work show that the employed DJ corrugated 2D additive represents an interesting material to improve the characteristics of a standard 3D perovskite.

## Conflicts of interest

There are no conflicts to declare.

## Supplementary Material

NA-005-D2NA00942K-s001
