# Supplementary material for: Role of a corrugated Dion–Jacobson 2D perovskite as an additive in 3D MAPbBr3 perovskite-based light emitting diodes
Source: Nanoscale Adv. 2023 Mar 29;5(9):2508–16. doi: 10.1039/d2na00942k (PMC10153086; doi:10.1039/d2na00942k)
Supplement: NA-005-D2NA00942K-s001 [file NA-005-D2NA00942K-s001.pdf]

Electronic Supplementary Material (ESI) for Nanoscale Advances.
This journal is © The Royal Society of Chemistry 2023

SUPPORTING INFORMATION

Role of corrugated Dion-Jacobson 2D perovskite as additive in 3D $MAPbBr_3$ perovskite-based light emitting diodes

C. T. Prontera, D. Taurino A. Coriolano, A. Maggiore, M. Pugliese, R. Giannuzzi, F. Mariano, S. Carallo, A. Rizzo, G. Gigli, L. De Marco, V. Maiorano

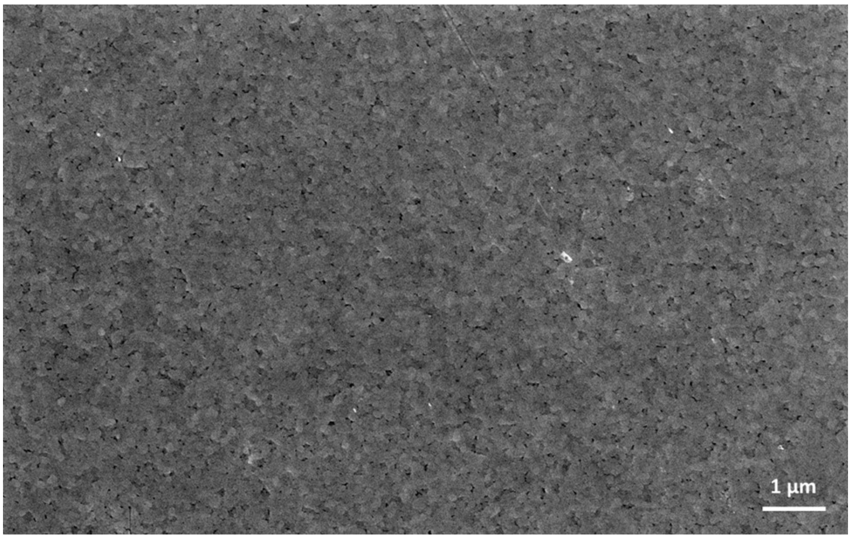

Figure S1: SEM picture of $MaPbBr_3$ 3D perovskite

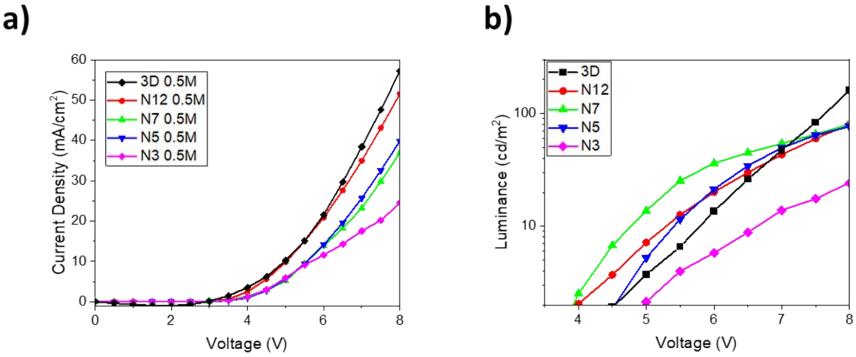

Figure S2: a) current density vs voltage curves for LED devices obtained with 3D, N12, N7, N5 and N3 thin films (0.5M); b) luminance vs voltage curves for LED devices obtained with 3D, N12, N7, N5 and N3 thin films (0.5M)

Table S1: Luminance, Current Efficiency and EQE of LED devices obtained with 3D, N12, N7, N5 and N3 thin films (0.5M)

	**Lum. (cd/m²)**	**CE (cd/A)**	**EQE (%)**
**3D**	160 @ 8V	0.28 @ 8V	0.08 @ 8 V
**N12**	80 @ 8V	0.16 @ 8V	0.04 @ 8V
**N7**	80 @ 8V	0.22 @ 8V	0.07 @ 8V
**N5**	77 @ 8V	0.19 @ 8V	0.07 @ 8V
**N3**	24 @ 8V	0.10 @ 8V	0.03 @ 8V

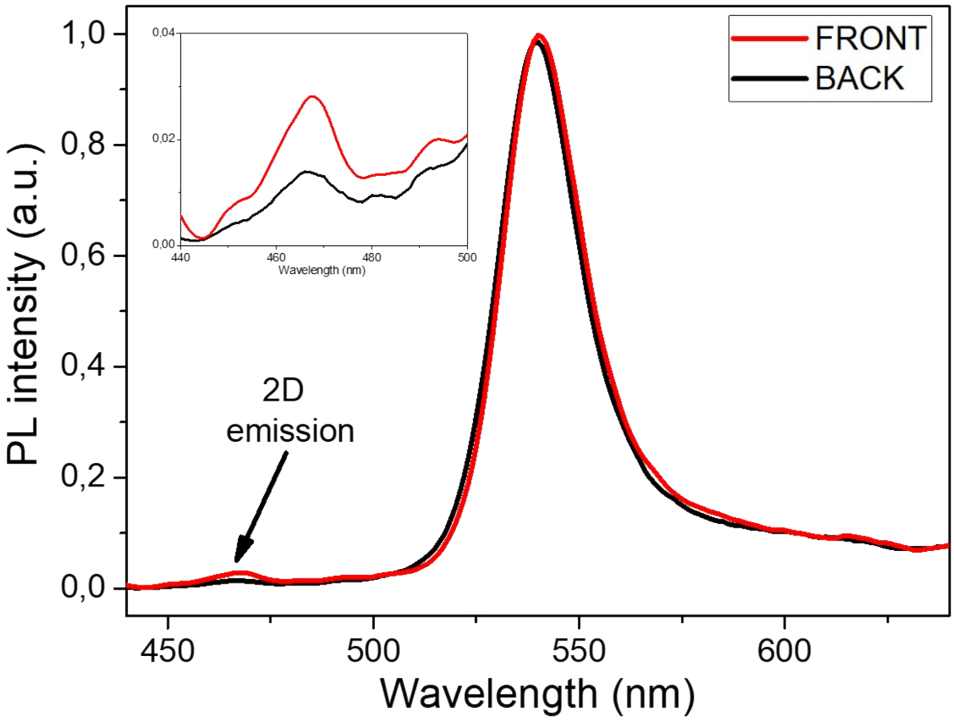

Figure S3: PL spectra of the 3D/2D bilayer from the "front" and the "back"

Table S2: Summary table related to employment of 2D DJ perovskites in LED devices

**REF**	**Perovskite structure**	**Device architecture**	**EQE (%)**	**Operational stability (T50)**
1	BAB-$FAPbI_3$ (BAB = 1,4-bis(aminomethyl)benzene)	ITO/ZnO/PEIE/perovskite/TFB/MoO_3/Al	5.2%	100 h
2	EDBE$(MAPbBr_3)_{n-1}PbBr_4$ (EDBE: 2,2-(ethylenedioxy)bis (ethylammonium))	ITO/PEDOT:PSS/perovskite/TPBi/LiF/Al	1.06	n.a.
3	$PbBr_2$:MABr:BDADBr (1,4-diaminobutane hydrobromide)	ITO/PEDOT:PSS/perovskite/TmPyPB/CsF/Al	1.1	3500 s
4	PbI_2: CsI: MAI: mXDA (m-xylylenediamine)	ITO/ZnO/PEIE/perovskite/TFB/MoO_3/Ag	12%	n.a.

5	$BDAFA_{n-1}PbnI_{3n+1}$ (BAD = 1,4-butanediamine)	ITO/ZnO/PEIE/perovskite/TFB/MoO_3/Au	9	189.4 h
6	DPDA-$CsPb(Br/Cl)_3$ (DPDA = N,Ndimethyl-1,3-propanediamine)	ITO)/modified-(PEDOT:PSS)/perovskites/TPBi)/(Liq)/aluminum (Al)	2.65	n.a.
7	$(PDMA)FA_2Pb_3X_{10}$ (PDMA = p-xylylenediamine)	ITO/ZnO/PEIE/perovskite/TFB/MoO_3/Au	7.1	n.a.
This work	$MAPbBr_3$/α(DMEN)$PbBr_4$ (α(DMEN) = (dimethylamino)ethylamine)	ITO/PEDOT:PSS/perovskite/BPhen/BPhen:Cs/Ag	0.27	1400 s

T50 = time that the device takes for the luminance to drop to half its initial value during constant applied voltage

1 Y. Shang, Y. Liao, Q. Wei, Z. Wang, B. Xiang, Y. Ke, W. Liu and Z. Ning, *Sci. Adv.*, 2019, **5**, 1–9.

2 C. H. Chen, Z. Li, Q. Xue, Y. A. Su, C. C. Lee, H. L. Yip, W. C. Chen and C. C. Chueh, *Org. Electron.*, , DOI:10.1016/j.orgel.2019.105400.

3 L. He, Z. Xiao, X. Yang, Y. Wu, Y. Lian, X. Peng and X. Yang, *J. Mater. Sci.*, 2020, **55**, 7691–7701.

4 Y. Xu, W. Xu, Z. Hu, J. A. Steele, Y. Wang, R. Zhang, G. Zheng, X. Li, H. Wang, X. Zhang, E. Solano, M. B. J. Roeffaers, K. Uvdal, J. Qing, W. Zhang and F. Gao, *J. Phys. Chem. Lett.*, 2021, **12**, 5836–5843.

5 K. H. Ngai, Q. Wei, Z. Chen, X. Guo, M. Qin, F. Xie, C. C. S. Chan, G. Xing, X. Lu, J. Chen, K. S. Wong, J. Xu and M. Long, *Adv. Opt. Mater.*, 2021, **9**, 1–10.

6 Y. Liu, L. K. Ono, G. Tong, T. Bu, H. Zhang, C. Ding, W. Zhang and Y. Qi, *J. Am. Chem. Soc.*, 2021, **143**, 19711–19718.

7 H. Yang, J. Tang, L. Deng, Z. Liu, X. Yang, Z. Huang, H. Yu, K. Wang and J. Li, *Phys. Chem. Chem. Phys.*, 2022, **24**, 7969–7977.
